# Probabilistic microsimulation to examine the cost-effectiveness of hospital admission screening strategies for carbapenemase-producing enterobacteriaceae (CPE) in the United Kingdom

**DOI:** 10.1007/s10198-021-01419-5

**Published:** 2021-12-21

**Authors:** Sarkis Manoukian, Sally Stewart, Stephanie J. Dancer, Helen Mason, Nicholas Graves, Chris Robertson, Alistair Leonard, Sharon Kennedy, Kim Kavanagh, Benjamin Parcell, Jacqui Reilly

**Affiliations:** 1 Yunus Centre for Social Business and Health, Glasgow Caledonian University, Glasgow, Scotland UK; 2 Safeguarding Health Through Infection Prevention Research Group, Glasgow Caledonian University, Glasgow, Scotland UK; 3 Department of Microbiology, Hairmyres Hospital, NHS Lanarkshire and School of Applied Sciences, Edinburgh Napier University, Edinburgh, Scotland UK; 4 Duke-NUS Medical School, Singapore, Singapore; 5 Department of Mathematics and Statistics, University of Strathclyde, Glasgow, Scotland UK; 6 NHS Greater Glasgow and Clyde, Glasgow, Scotland UK; 7 Information Services Division, Public Health Scotland, Edinburgh, Scotland UK; 8 Medical Microbiology, NHS Tayside, Ninewells Hospital and School of Medicine, Dundee, Scotland UK

**Keywords:** Health Economics, Screening programmes, Healthcare-associated infection, Carbapenemase-producing-Enterobacteriaceae, Microsimulation, National Health Service

## Abstract

**Background:**

Antimicrobial resistance has been recognised as a global threat with carbapenemase- producing-Enterobacteriaceae (CPE) as a prime example. CPE has similarities to COVID-19 where asymptomatic patients may be colonised representing a source for onward transmission. There are limited treatment options for CPE infection leading to poor outcomes and increased costs. Admission screening can prevent cross-transmission by pre-emptively isolating colonised patients.

**Objective:**

We assess the relative cost-effectiveness of screening programmes compared with no- screening.

**Methods:**

A microsimulation parameterised with NHS Scotland date was used to model scenarios of the prevalence of CPE colonised patients on admission. Screening strategies were (a) two-step screening involving a clinical risk assessment (CRA) checklist followed by microbiological testing of high-risk patients; and (b) universal screening. Strategies were considered with either culture or polymerase chain reaction (PCR) tests. All costs were reported in 2019 UK pounds with a healthcare system perspective.

**Results:**

In the low prevalence scenario, no screening had the highest probability of cost-effectiveness. Among screening strategies, the two CRA screening options were the most likely to be cost-effective. Screening was more likely to be cost-effective than no screening in the prevalence of 1 CPE colonised in 500 admitted patients or more. There was substantial uncertainty with the probabilities rarely exceeding 40% and similar results between strategies. Screening reduced non-isolated bed-days and CPE colonisation. The cost of screening was low in relation to total costs.

**Conclusion:**

The specificity of the CRA checklist was the parameter with the highest impact on the cost-effectiveness. Further primary data collection is needed to build models with less uncertainty in the parameters.

**Supplementary Information:**

The online version contains supplementary material available at 10.1007/s10198-021-01419-5.

## Introduction

Antimicrobial resistance has been recognised by the World Health Organisation as a global threat to public health, with carbapenemase-producing-Enterobacteriaceae (CPE) identified as a major problem [1–3]. Enterobacteriaceae are Gram-negative bacteria capable of causing a range of infections particularly in patients who are already unwell [3]. Enterobacteriaceae include *Escherichia coli* and *Klebsiella pneumoniae*, both of which contribute towards Healthcare-Associated Infection (HAI) and community infection. There are similarities between COVID-19 and CPE as asymptomatic patients may be colonised with CPE and act as a source of transmission to others via hands, body fluids, equipment and the environment. When CPE infection occurs, there are limited treatment options leading to poor patient outcomes and increased costs of care due to additional care and longer length of stay (LOS) in hospital [4–7].

NHS Scotland has identified more cases of CPE each year since they were first reported in 2003 [8]. The estimated incidence of carbapenemase-producing-organisms (CPO) (89.8% of which were CPE in 2018) increased fivefold between 2013 and 2017 [8] and varies substantially across the UK regions [9]. The risk of morbidity and mortality from these infections is high because the causative organisms are already resistant to last-resort antibiotics. Given the lack of treatment options, it is important to focus on prevention through screening and testing programmes. Previous work has shown that the introduction of a CPE screening programme using admission polymerase chain reaction (PCR) testing in intensive care units was cost-effective [10]. Lapointe Shaw et al. [11] modelled the implementation of CPE screening in Canada and found that there was only a modest benefit due to the current low prevalence of CPE colonisation. They concluded that screening of all admissions using PCR or culture was cost-effective due to the morbidity and mortality of CPE infections [11].

Moloney et al. [12] used a decision tree to show that PCR-based screening of high-risk patients offers savings to the health service comparing with culture-based tests. These previous studies are based on static modelling approaches which do not allow explicit modelling of infectious disease dynamics.

In the United Kingdom, the low incidence of CPE has led to the introduction of a clinical risk assessment (CRA) checklist, which identifies patients who may benefit from microbiological screening [13]. There remains, however, a lack of evidence on the clinical and economic benefits of different screening approaches. The current screening programme in NHS Scotland is a type of targeted screening programme that prioritises colonised patients for isolation to prevent additional transmission events. In Scotland, since 2013 a positive CRA is followed by a culture-based microbiological test for CPE. The CRA currently comprises of three questions, with patients receiving a test if they answer ‘yes’ to any of these questions: Has the patient been an inpatient in a hospital abroad? Have they received dialysis abroad? Have they been a close contact with a person colonised or infected with CPE?

This study uses NHS Scotland data and builds on recent cost-effectiveness modelling in international settings to assess the cost per quality-adjusted life year (QALY) gained for different CPE screening strategies in the NHS. This is the first study, to our knowledge, of CPE screening which is based on a probabilistic microsimulation with a dynamic transmission modelling approach. Our approach gives more flexibility in how patients, timing of events, the disease process and interventions are modelled than traditional static cohort-based Markov models [10, 11, 14, 15]. The cost and impact of screening on key outcomes such as in-hospital cross-transmissions, number of infections and deaths are also presented. Different scenarios of the the prevalence of CPE colonised patients on admission are modelled to examine the cost-effectiveness of different screening strategies when compared to no-screening, as these epidemiological conditions change.

## Methods

A Markov microsimulation model of individual patients was used to simulate pathways through a hospital screening programme and to estimate the relative cost–effectiveness of no-screening and screening strategies. The model was implemented using decision analysis software (TreeAge Pro) [16]. The model incorporates parameter uncertainty directly with outcomes depending on patient age and chance as would happen in a real setting. A daily cycle was chosen since in this population hospital stays are short with many daily admissions and discharges and due to the fast-paced nature of the screening programme, CPE colonisation and disease process. The model simulated a period of three years to capture long-term outcomes relevant to a CPE screening programme. The simulation begins with 600 simulated patients with 80 simulated patients admitted daily to the hospital. We present results from probabilistic analyses based on 500 model iterations, with random re-samples from parameter distributions, in four different scenarios of prevalence on admission CPE colonised patients.

### Strategies

Admission screening was modelled for a flow of patients admitted into NHS acute hospitals for inpatient care. Current policy for CPE screening has two steps: first, an initial CRA checklist that identifies patients who are more likely (high-risk) to be CPE colonised, and secondly, refers those identified for a microbiological test. Our model tested different screening strategies following CRA and universal screening. There are two types of microbiological tests available, the first is phenotypic testing using standard or chromogenic agar culture and the second is genotypic testing or PCR. Culture is low cost but may take 2–4 days to return a result and PCR is higher cost but returns results usually within a day [17].

The modelled strategies combine the above and were developed through consultations with representatives from NHS Scotland and infection prevention and control (IPC) clinicians.

They are as follows:

1. No-screening, but isolate patients identified with CPE infection.
2. CRA checklist followed by a screening test (culture/phenotypic) for CPE for those identified as high risk by the CRA.
3. CRA checklist followed by a screening test (PCR/genotypic) for CPE for those identified as high risk by the CRA.
4. All inpatient screening (culture/phenotypic) on admission (universal screening).
5. All inpatient screening (PCR/genotypic) on admission (universal screening).

Strategy 1 did not involve screening on admission; however, if a patient developed signs of infection they were isolated from the general population. In this strategy, we did not take into account any costs that would accompany CPE infection other than isolation and treatment costs. Strategies 2 and 3 were based on the current CPE screening programme in NHS Scotland [18]. In practice, some hospitals employ a combination of these strategies, by using both culture and PCR testing. Strategies 4 and 5 are universal screening strategies that are not currently applied in NHS Scotland but given the epidemiology of CPE could be revisited in the future.

**Fig. 1:**
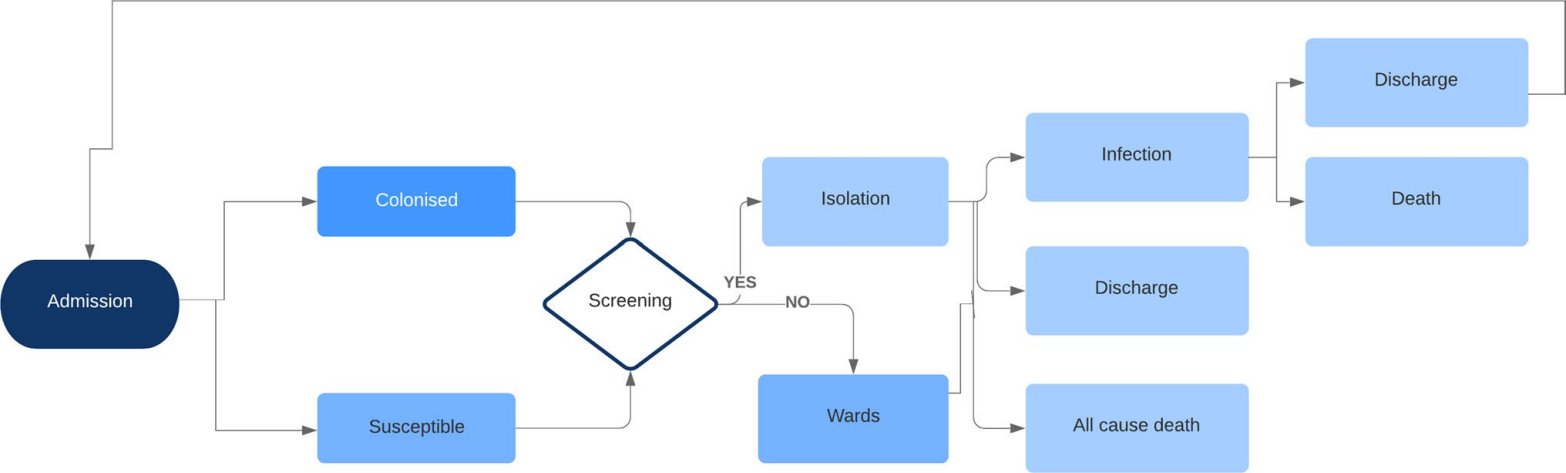
Simplified diagram of model structure. Susceptible refers to the patient carrying no CPE. Screening refers to clinical risk assessment followed by lab screening method with culture or PCR

A simplified version of the screening strategies (2–5) in the model is presented in Fig. 1. Supplementary materials (SM) 1 show the full state transition paths via the health states for all the strategies of the model.

### Model conceptualisation

**Fig. 2:**
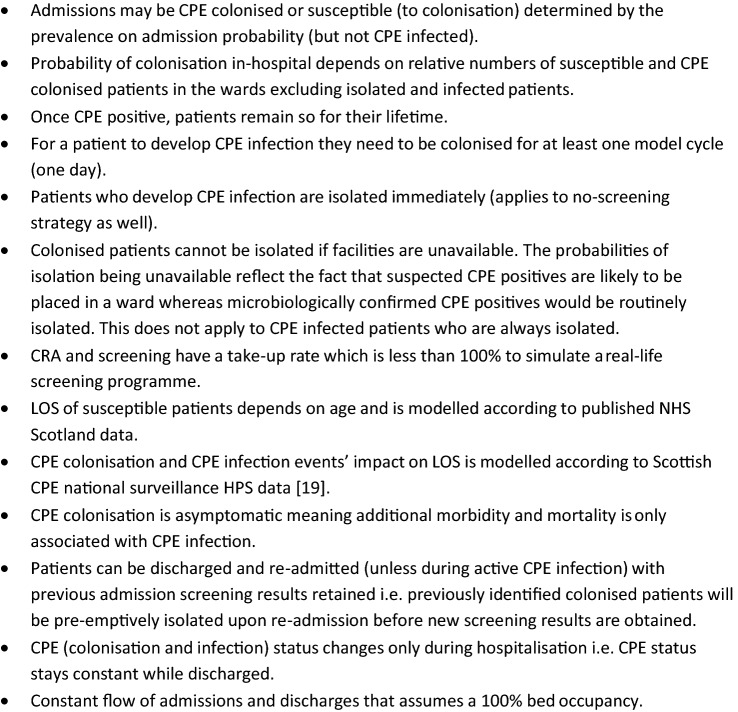
Key model features and assumptions

In this simulation individual patients progress through the model in parallel to allow interactions between patients. Each patient remained in the model until death, or the end of the three-year period, since re-admission is modelled explicitly [19]. Key model features and assumptions are presented in Fig. 2.

The model was based on a two-stage disease process – CPE colonisation and CPE infection. CPE colonisation is asymptomatic and not every colonised patient develops infection. A colonised individual may develop two types of infections either a localised form of CPE infection defined as “CPE Local” which can be a single organ space infection or severe infection defined as “CPE Systemic” which can be bloodstream infection or sepsis. The simulation allows for false positives/negatives in the CRA and microbiological tests to occur and these were modelled as a probability related to the sensitivity and specificity parameter distributions. A false-positive result in the screening process can lead to unnecessary testing and isolation of patients.

### Model input parameters

Data has been derived from the literature and NHS Scotland. NHS Scotland sources included Information Services Division (ISD) publicly available data and surveillance data. Approvals were received for aggregate surveillance data held at Health Protection Scotland (HPS) for all CPE-positive patients from 2013 to 2017 linked to acute hospital inpatient records held by ISD Scotland. SM 2 shows the value, plausible range, distribution and source of all data items used in this model. The prevalence of CPE colonised patients on admission is important for the cost-effectiveness of any screening programme and according to HPS surveillance data this could be as low as 1 in 10,000 admissions. The epidemiology of CPE is constantly changing in the UK and other countries [3, 8]. Given the variability of CPE in the UK and Europe the analysis was conducted in the following prevalence on admission scenarios: 1 in 10,000, 1 in 1,000, 1 in 500 and 1 in 100 patients.

Key parameters included the sensitivity and specificity of CRA and microbiological tests. One hospital study has published a percentage of CPE colonised patients with similar risk factors to the Scottish CRA questions [20]. Based on these numbers we assumed that CRA has poor sensitivity but fairly good specificity. There was better evidence for sensitivity and specificity of phenotypic and genotypic microbiological tests, which is very high for both [21, 22]. One difference between these tests was how long it took to get results, with PCR being faster and more accurate but also more costly than culture (see SM 2).

LOS of patients with CPE colonisation and infection was modelled using surveillance HPS data. LOS of susceptible inpatients was determined using publicly available NHS Scotland data [23]. Inpatient stay was modelled to depend on age with older patients having longer LOS. In this model patients have a baseline expected LOS depending on their age and an additional LOS if they become CPE colonised or develop CPE infection. For CPE-positive patients LOS is determined by expected stay, based on HPS data, minus time already spent in hospital (i.e. before developing colonisation and/or infection). Mortality due to CPE local and systemic infection has been reported to be between 12.4 and 73.1% [24, 25]. Some studies suggest up to 100% mortality for specific organisms or groups of patients [26].

#### Infection dynamics

A susceptible-colonised equation was employed to calculate the transmission probability for each susceptible patient in the model [11]. Patients who have CPE infection are excluded as they are assumed to have been placed in isolation. In-hospital transmission also depends on the basic reproductive rate of CPE [27] which has been estimated to be between 1.5 and 3 for CPE patients (see SM 2) [28].

#### Health state utility values

Health state utility values were obtained from the literature as information from CPE patients was not available. One study with a cost-utility analysis of CPE screening used values from MRSA patients [11]. We used a similar approach by taking values from two MRSA screening studies [14, 29], and utility values for the general hospital population from other sources [30–33]. As none of these data represented patients with a CPE infection, variability in the utility values was allowed as reflected by the distributions of these parameters. It was assumed that asymptomatic colonisation does not impact on quality of life as opposed to symptomatic CPE infection which was expected to have substantial impact.

#### Resource use and costs

Ward and ICU bed-day costs were calculated by taking a weighted average of the cost from all teaching and general hospital beds in NHS Scotland [34]. This model included one-off and daily costs. Screening costs included CRA and microbiological tests as one-off costs that occur on admission and are not repeated. PCR costs also included culture costs since live organisms are needed for subsequent susceptibility testing. In reality, this does not necessarily impact on the speed of PCR or how quickly associated decisions are taken following a positive test. Contact precautions and IPC costs are calculated on a daily basis and depend on LOS. CPE infection imposes daily treatment and IPC costs and treatment costs were based on published antibiotic prescribing [35–37]. Antibiotics appropriate for CPE infection may cause severe renal toxicity and require a monitoring regimen [35]. An initial one-off toxicity test cost was applied to CPE-infected patients followed by a daily average cost from the second day of treatment.

All CPE systemic infections are assumed to require intensive care, which has a more costly bed-day than LOS in wards making systemic infections the most expensive health state in the model. Isolation increases daily costs by requiring a daily IPC routine [38]. Patients with local and systemic infections have identical IPC and treatment costs and only differ in the location of treatment (ward vs ICU) and mortality. All costs were reported in 2019 UK pounds due to cost data being prospectively collected in that year and reported using an NHS perspective. Discounting was applied to all costs and outcomes at 3.5% [39].

## Results

Results of the probabilistic analyses are presented according to the four epidemiologic scenarios. Isolation-related patient outcomes are presented in Table 1, CPE colonisation and infection patient outcomes are presented in Table 2 and cost-effectiveness results are presented in Table 3.

**Table 1:** Isolation outcomes, by screening strategy and prevalence of CPE colonised on admission

Prevalence of CPE positive on admission	Strategy	CPE positive bed-days not in isolation^1^	Patients inappropriately isolated^2^	Total bed-days inappropriate isolation^3^	Patients appropriately isolated^4^	Total bed-days appropriate isolation^5^
1 in 10,000	No screening (Strategy 1)	678	N/A	N/A	N/A	N/A
1 in 10,000	CRA Culture (Strategy 2)	127	2,161	8,163	35	351
1 in 10,000	CRA PCR (Strategy 3)	118	1,452	3,518	40	398
1 in 10,000	Universal culture (Strategy 4)	83	13,983	68,755	41	413
1 in 10,000	Universal PCR (Strategy 5)	77	5,599	28,352	44	407
1 in 1,000	No screening (Strategy 1)	6,690	N/A	N/A	N/A	N/A
1 in 1,000	CRA Culture (Strategy 2)	1,267	2,141	8,094	366	3,670
1 in 1,000	CRA PCR (Strategy 3)	1,184	1,453	3,542	367	3,705
1 in 1,000	Universal culture (Strategy 4)	799	13,985	68,858	405	4,072
1 in 1,000	Universal PCR (Strategy 5)	673	5,546	28,114	414	3,920
1 in 500	No screening (Strategy 1)	13,526	N/A	N/A	N/A	N/A
1 in 500	CRA Culture (Strategy 2)	2,729	2,091	7,826	717	7,113
1 in 500	CRA PCR (Strategy 3)	2,495	1,451	3,540	720	7,283
1 in 500	Universal culture (Strategy 4)	1,645	13,441	65,966	789	7,825
1 in 500	Universal PCR (Strategy 5)	1,351	5,697	28,878	865	8,151
1 in 100	No screening (Strategy 1)	65,293	N/A	N/A	N/A	N/A
Prevalence of CPE positive on admission	Strategy	CPE positive bed-days not in isolation^1^	Patients inappropriately isolated^2^	Total bed-days inappropriate isolation^3^	Patients appropriately isolated^4^	Total bed-days appropriate isolation^5^
1 in 100	CRA Culture (Strategy 2)	12,652	2,125	8,047	3,607	36,021
1 in 100	CRA PCR (Strategy 3)	12,115	1,427	3,471	3,663	36,859
1 in 100	Universal culture (Strategy 4)	7,750	13,842	68,139	3,940	39,279
1 in 100	Universal PCR (Strategy 5)	7,020	5,522	28,000	4,236	39,712

Notes (Reported means from 500 iterations of probabilistic sensitivity analyses over the 3 year period and 88,200 unique patients) 1 Non-isolated CPE positive bed-days

CPE false-positive number of patients

CPE false-positive bed-days

CPE true-positive number of patients

CPE true-positive bed-days

**Table 2:** CPE colonisation and infection patient outcomes, by screening strategy and prevalence of CPE

Prevalence of CPE positive on admission	Strategy	Cross- transmissions^1^	CPE local infections^2^	CPE systemic infections^3^	Deaths due to local infection^4^	Deaths due to systemic infection^5^
1 in 10,000	No screening (Strategy 1)	5.86	19.90	1.54	1.84	0.60
1 in 10,000	CRA Culture (Strategy 2)	0.96	14.72	0.96	1.06	0.44
1 in 10,000	CRA PCR (Strategy 3)	1.04	16.60	1.30	1.42	0.52
1 in 10,000	Universal culture (Strategy 4)	0.64	14.58	1.20	1.34	0.44
1 in 10,000	Universal PCR (Strategy 5)	0.46	15.90	1.24	1.50	0.60
1 in 1,000	No screening (Strategy 1)	53.60	197.38	15.80	16.56	6.96
1 in 1,000	CRA Culture (Strategy 2)	10.48	146.20	11.20	11.60	4.82
1 in 1,000	CRA PCR (Strategy 3)	9.16	144.66	11.50	11.82	5.48
1 in 1,000	Universal culture (Strategy 4)	6.46	140.28	10.68	12.04	4.76
1 in 1,000	Universal PCR (Strategy 5)	5.18	138.60	10.74	11.34	4.66
1 in 500	No screening (Strategy 1)	113.52	405.98	30.90	34.74	13.38
1 in 500	CRA Culture (Strategy 2)	21.62	297.50	23.00	25.48	10.82
1 in 500	CRA PCR (Strategy 3)	20.08	290.80	22.94	24.38	10.72
1 in 500	Universal culture (Strategy 4)	13.56	281.44	23.00	24.26	9.94
1 in 500	Universal PCR (Strategy 5)	10.46	281.46	21.86	23.34	9.60
1 in 100	No screening (Strategy 1)	497.58	1,953.84	152.00	162.10	68.32
1 in 100	CRA Culture (Strategy 2)	98.02	1,460.92	113.20	126.28	50.42
1 in 100	CRA PCR (Strategy 3)	92.94	1,453.80	111.74	124.26	50.00
1 in 100	Universal culture (Strategy 4)	59.56	1,404.26	109.60	119.10	49.48
1 in 100	Universal PCR (Strategy 5)	52.96	1,397.84	110.82	116.38	48.80

Notes (Reported means from 500 iterations of probabilistic sensitivity analyses over the 3 year period and 88,200 unique patients) 1 Cross-transmissions are CPE colonisations that happen in the hospital environment

CPE local infections in hospital

CPE systemic infections in hospital

Number of deaths due to CPE local infection

Number of deaths due to CPE systemic infection

Screening strategies reduced in-hospital cross-transmission and infection, with universal screening having the greatest effect, when compared with no-screening for all scenarios. (see Table 2). In all prevalence scenarios CRA screening strategies reduced transmissions of CPE colonisation by approximately 81% from baseline “no-screening” strategy. Universal screening reduced transmissions by a further 40–43% compared to CRA screening (or about 89% from baseline “no-screening” strategy). These results followed the same pattern for isolation outcomes since cross-transmission directly depends upon non-isolated bed- days. All screening strategies resulted in reduced numbers of CPE infections (27–32%)

**Table 3:** Probability of cost-effectiveness, incremental costs, effectiveness and screening costs, by the prevalence of CPE colonised on admission

Prevalence of CPE positive on admission	Cost-effective strategies^1^	Non-cost- effective strategies^1^	Probability of cost- effectiveness^2^	Incremental costs^3^	Incremental effectiveness^4^	CRA costs^5^	Microbiological test costs^6^
1 in 10,000	No screening (Strategy 1)		40%			N/A	N/A
1 in 10,000		CRA Culture (Strategy 2) (£123,877/QALY)	25%	£859,071	7	£748,370	£108,852
1 in 10,000		CRA PCR (dominated) (Strategy 3)	24%	£1,252,909	− 22	£749,747	£612,388
1 in 10,000		Universal culture (Strategy 4) (£940,888/QALY)	10%	£5,011,291	11	N/A	£1,211,829
1 in 10,000		Universal PCR (dominated) (Strategy 5)	1%	£8,076,649	4	N/A	£6,960,778
1 in 1,000	No screening (Strategy 1)		36%			N/A	N/A
1 in 1,000		CRA Culture (Strategy 2) (£214,621/QALY)	28%	£795,917	4	£748,473	£112,001
1 in 1,000		CRA PCR (Strategy 3) (£83,203/QALY)	28%	£948,729	11	£750,044	£633,894
1 in 1,000		Universal culture (Strategy 4) (dominated)	6%	£4,869,327	− 9	N/A	£1,215,224
1 in 1,000		Universal PCR (Strategy 5) (£676,166/QALY)	2%	£7,873,538	12	N/A	£6,973,598
1 in 500	CRA Culture (Strategy 2)		45%			£744,954	£114,381
1 in 500		No screening (Strategy 1) (dominated)	26%	£215,136	− 16	N/A	N/A
1 in 500		CRA PCR (Strategy 3) (£994,198/QALY)	24%	£342,830	0	£746,102	£647,753
Prevalence of CPE positive on admission	Cost-effective strategies^1^	Non-cost- effective strategies^1^	Probability of cost- effectiveness^2^	Incremental costs^3^	Incremental effectiveness^4^	CRA costs^5^	Microbiological test costs^6^
1 in 500		Universal culture (Strategy 4) (dominated)	3%	£3,754,032	− 12	N/A	£1,223,968
1 in 500		Universal PCR (Strategy 5) (dominated)	2%	£6,875,555	− 12	N/A	£7,025,426
1 in 100	CRA Culture (Strategy 2)		34%			748,082	144,657
1 in 100		CRA PCR (Strategy 3) (£55,915/QALY)	38%	£517,726	9	749,885	823,072
1 in 100		Universal culture (Strategy 4) (£87,205/QALY)	20%	£3,519,953	40	N/A	1,242,347
1 in 100		No screening (Strategy 1) (dominated)	6%	£6,356,878	− 41	N/A	N/A
1 in 100		Universal PCR (Strategy 5) (£658,558/QALY)	2%	£6,613,977	10	N/A	7,125,937

Notes (Reported means from 500 iterations of probabilistic sensitivity analyses over the 3 year period and 88,200 unique patients)

An ICER of less than £30,000 per QALY is deemed to be cost-effective; an ICER of more than £30,000 (the upper limit of the usual willingness-to-pay range of the NICE threshold guidance) is deemed to not be cost-effective[40]. ICERs are shown in relation to cost-effective strategy. ICERs are not shown if incremental benefit is negative. QALY = quality-adjusted life-year. ICERs are shown in 2019 UK pounds, average inflation in 2020/2021 was 1.25%.

Frequency strategy is optimal at the £30,000 willingness-to-pay per QALY threshold over 500 model iterations.

Table orders strategies by incremental costs. Incremental costs are reported in comparison to the least costly strategy. 4 Quality-adjusted life years (QALYs)

Clinical risk assessment costs

Microbiological test costs

Results in Table 1 show that compared with the baseline strategy of no-screening, screening strategies (with CRA or universal) were effective in identifying CPE-positive patients and isolating them from the general hospital population. This became more obvious in higher prevalence on admission scenarios. Among the screening strategies universal screening had the most, but also inappropriate, isolation days, and the fewest non-isolated CPE-positive days when compared with CRA screening (see Table 1). PCR screening had fewer non- isolated CPE-positive days, fewer inappropriate isolations and more appropriate isolations than culture screening strategies. Screening strategies reduce non-isolated bed-days of CPE positive patients by more than 80% in comparison with no-screening. PCR can reduce non- isolated bed-days by a further 40% when compared with culture testing (approximately 89% reduction from the baseline no-screening strategy). These results show that, compared with CRA screening, universal screening increases identification of CPE-positive patients by approximately 14% but at the same time increases inappropriate isolation by about 74%.

PCR, when compared with culture, reduces inappropriate isolation by about 60% and increases identification of CPE-positive patients by between 2 to 8% depending on the scenario.

While screening strategies target cross-transmission, the incidence of CPE infection is driven by many other factors. Therefore, even though all strategies reduced CPE positive bed-days in wards (non-isolated) and cross-transmission the differences between strategies in terms of CPE infections were small for all epidemiological scenarios.

Screening costs vary according to strategy starting from approximately £860,000 (strategy 2) to £7 million pounds (strategy 5) in the three-year period (88% difference in screening costs). To put these in a per patient context these costs vary between £10 and £80 per patient treated in hospital during this period. The least costly strategy, in terms of screening costs, was targeted screening (CRA) with culture and the most expensive was universal screening with PCR (see Table 3). Screening costs were a small portion of total costs, from approximately 1.5% to 3% of total inpatient costs (see SM 3). Differences in incremental costs were mostly driven by screening costs in the low prevalence scenarios but in higher prevalence scenarios this was due to reductions in CPE infections. This made screening strategies cost-saving when compared with no-screening. Differences in incremental QALYs were very small and ranged at around 0.02% of total effectiveness (see SM 3).

Probability of cost-effectiveness did not exceed 50% for any strategy in all scenarios. In the low prevalence scenarios at willingness-to-pay values at the top of the NICE threshold guidance (£30,000 per QALY gained), the no-screening strategy had the highest probability of cost-effectiveness (40–36%), followed by CRA screening (25–28%). CRA screening strategies were most likely to be cost-effective in the higher prevalence scenarios. Among this culture was the most likely to be the cost-effective microbiological test in the 1 in 1,000 prevalence scenario. PCR had the highest probability of cost-effectiveness in the 1 in 100 scenario but differences between the CRA strategies were small in every scenario. The two universal screening strategies were unlikely to be cost-effective in all prevalence scenarios.

**Fig. 3:**
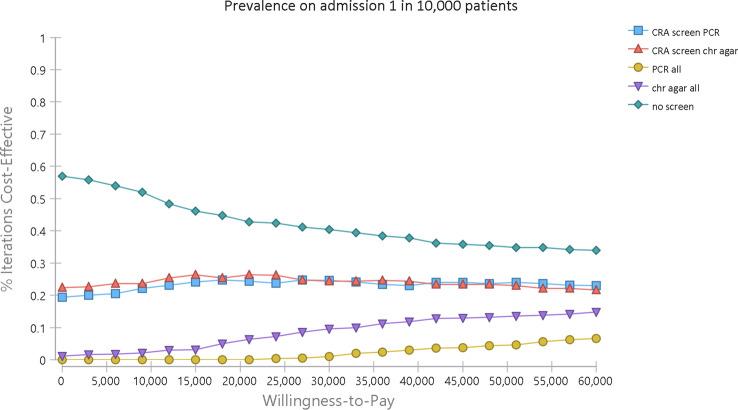
Cost-effectiveness acceptability curve at CPE colonised prevalence on admission 1 in 10,000 patients. “CRA screen PCR” and “CRA screen chr agar” on figure refer to strategy 3 and 2, respectively. “PCR all” and “chr agar all” refer to strategy 5 and 4, respectively. “no screen” refers to strategy 1

The cost effectiveness acceptability curves (CEAC) for a range of willingness-to-pay per QALY gained thresholds were also estimated [41]. These present the probability that a given strategy is more cost- effective than the alternatives for a range of thresholds [41]. We present the CEAC in the scenario of 1 in 10,000 patients in Fig. 3 and the other scenarios can be found in SM 3.

Strategy 1 (no-screening) was the optimal strategy followed by the two CRA screening strategies for prevalence on the admission of 1 in 10,000. The likelihood of cost-effectiveness for the two CRA strategies was around 25% along the NICE recommended willingness-to-pay range (£20,000-£30,000 per QALY gained). Strategy 4 (universal screening with culture) had a 10% probability of cost-effectiveness at the top of the NHS willingness-to-pay range.

Strategy 5 (Universal screening with PCR) was not likely to be cost-effective at this level of prevalence.

## Discussion

This is the first study, to our knowledge, to use a dynamic patient-level simulation to model the relative cost-effectiveness of screening strategies to reduce the transmission and the adverse health outcomes associated with CPE. Screening for CPE is important because these pathogens are resistant to last-resort antibiotics making hospital outbreaks very burdensome in terms of morbidity, mortality and cost of care [6, 7]. Patient outcomes were modelled using actual observed NHS Scotland data [19]. Microbiologically confirmed cases in the data suggest very low prevalence in Scotland but it is possible asymptomatic carriers were not screened making actual prevalence uncertain. Four epidemiological scenarios were presented since CPE prevalence varies between UK regions and may be higher than previously estimated [9, 37, 42]. The optimal screening strategy in the low prevalence scenarios is CRA culture screening (Strategy 2) of high-risk patients who are then isolated. As CPE prevalence on admission increases, the speed of patient placement decisions becomes more important and the CRA should be followed by PCR testing which is more costly and was modelled to provide faster results with fewer false positives.

The goal of CPE screening programmes is to identify and isolate colonised patients from the hospital population [18, 43]. CPE screening does not involve decolonisation or treatment of CPE positive patients and its only impact is through risk-reduction of in-hospital cross-transmission events through isolation of colonised patients. The benefit of CPE screening was to reduce the number of non-isolated CPE positive bed-days when compared with no- screening, which translated to a lower risk of acquisition of CPE for all susceptible patients. Given the low cost of targeted (CRA) screening and its ability to identify colonised patients we recommend implementing a targeted CPE screening program in hospitals. Infection prevention cannot always wait for interventions to be shown to be cost-effective when faced with new emerging threats so need to continue to protect patients. As with the COVID-19 pandemic, the current programme is designed to test and isolate until a treatment becomes available. Knowing more than the “tip of the iceberg” can inform prevention measures and control the spread of CPE [44]. The results show that in higher prevalence scenarios CRA strategies were optimal. Given the rise in the incidence of CPE worldwide and reported outbreaks in hospitals, the role of an admission screening programme is key to the prevention of nosocomial transmission and excess costs [7]. Universal screening with either microbiological test was not likely to be cost-effective in any of the scenarios. Other key outcomes such as the number of infections, reductions in cross- transmission and deaths follow the same pattern as the overall cost effectivenessresults.

PCR or rapid testing methods can release hospital resources for other uses and their use should be explored further [17, 45].

Markov models have been used extensively in health economic applications including in the evaluation of different HAI screening strategies [10, 11, 14, 15]. Our results complement those by Lapointe-Shaw et al. [11] who implemented a static cohort approach and found CPE screening to be cost-effective with higher prevalence on admission. Our study found that CPE screening becomes the optimal strategy at 1 in 500 patients admitted colonised where Lapointe-Shaw et al. found that this can happen earlier at 1 in 1,000 patients.

Lapointe-Shaw et al. implemented longer model cycles and a single screening strategy with a 100% take-up rate as opposed to our model, which used a dynamic approach and examined a range of screening strategies with an imperfect take-up rate. In our study, a patient-level simulation was selected because it allowed a probabilistic analysis with parameter uncertainty to be modelled into heterogenous patients who experience random events and interact with each other.

The microsimulation approach allowed explicit modelling of time with patients’ LOS in hospital depending on characteristics and events. The drawback of this approach was the heavy computational burden which limited the total number of model cycles and unique patients that could have been added to the model. The computational burden of this type of simulation needed to be balanced with a sufficiently large number of patients and horizon that produced stable results. To ensure results were not sensitive to these decisions we tested models with different combinations of patient numbers and horizons. The results in these checks (available upon request) were very similar to the analyses presented. Perhaps in future research, it will be possible to reprogramme this model using a more efficient programming language which will allow extending the number of simulated patients and model horizon.

There was substantial uncertainty in this model with the probability of cost-effectiveness of any one strategy rarely exceeding 40%, and similar results between strategies in terms of these probabilities. This was due to the information we used that either came from single studies or a relatively small number of CPE colonised and infected patients in NHS Scotland. The distributions of parameters were wide reflecting the small number of patients in the NHS data and published studies. Furthermore, the probabilistic nature of the modelled processes meant a diverse range of outcomes was possible with each model iteration. The level of uncertainty in our model was similar to Robotham et al. [40] who modelled MRSA screening in a probabilistic manner. As more high-quality studies on CPE screening and CPE infection are published, parameter uncertainty should be reduced. It should be noted that since these events are based on probabilities there will always be uncertainty associated with these models. Overall our results show that no-screening is the optimal strategy in low prevalence scenarios but given the high uncertainty of this result, probability less than 50%, the health system should not abandon CPE screening. Due to the very low cost of CRA the best choice among screening strategies is CRA with culture-based screening of high-risk patients. We cannot confirm the finding of Moloney et al. [12] since our model showed culture-based screening to be superior to PCR in terms of cost-effectiveness except at the higher prevalence scenario.

Our study has several limitations. Some model parameters were based on data from studies which may not have been representative of conditions in the UK. Utility values were not collected prospectively but derived from general and MRSA literature. We did not consider the delay between developing infection and appropriate patient management in the no-screening strategy or the fact that colonisation and isolation might have an impact on quality of life. CPE screening may further improve patient outcomes by increasing the speed of treatment with appropriate antibiotics [24, 46]. Therefore, the results presented in this study may be conservative estimates of the true cost-effectiveness of screening. This model involved microbiological tests that were idealised versions of their real-life counterparts. We did not consider initial capital costs to set up PCR testing and we assumed tests would be done in an existing central lab therefore the time-to-result included time to get to the facility and potential queues for processing.

Future epidemiological research is needed to identify risk factors so that the CRA checklist can be appropriately adjusted to minimise unnecessary microbiological testing of low-risk patients. Sensitivity analysis (see SM 3) showed that the specificity of the CRA was the parameter with the highest impact on the cost-effectiveness. A well-designed screening programme would result in a low number of false positives, which would otherwise consume limited isolation rooms, testing capacity and staff time. These results are relevant in these times of increased demand for screening services due to the COVID-19 pandemic. Further investigation is needed especially primary data collection studies to enhance understanding of CPE and improve our ability to build decision-analytic models with fewer limitations and uncertainty in the parameters.

## Data

Surveillance data of CPE patients and testing in Scotland were obtained from Health Protection Scotland (HPS) by making an application to the Public Benefit and Privacy Panel for Health and Social Care (PBPP) Application number 1819–0333.

## Supplementary Information

Below is the link to the electronic supplementary material.

Supplementary file1 (PDF 1279 KB)
