# Supplementary material for: Probabilistic microsimulation to examine the cost-effectiveness of hospital admission screening strategies for carbapenemase-producing enterobacteriaceae (CPE) in the United Kingdom
Source: Eur J Health Econ. 2021 Dec 21;23(7):1173–85. doi: 10.1007/s10198-021-01419-5 (PMC8689289; doi:10.1007/s10198-021-01419-5)
Supplement: Supplementary file 1 — Supplementary file1 (PDF 1279 KB) [file 10198_2021_1419_MOESM1_ESM.pdf]

<u>Title</u>*:*

Probabilistic Microsimulation to Examine the Cost-Effectiveness of Hospital Admission Screening Strategies for Carbapenemase Producing Enterobacteriaceae (CPE) in the United Kingdom

Journal: Eur J Health Econ

Supplementary Materials 1-3

Contents

1. State Transition Diagrams 3
2. Model Parameters 6
3. Additional Results 16
4. References 23

1. State Transition Diagrams

The state transition diagrams are identical for culture and PCR strategies so these are not shown separately

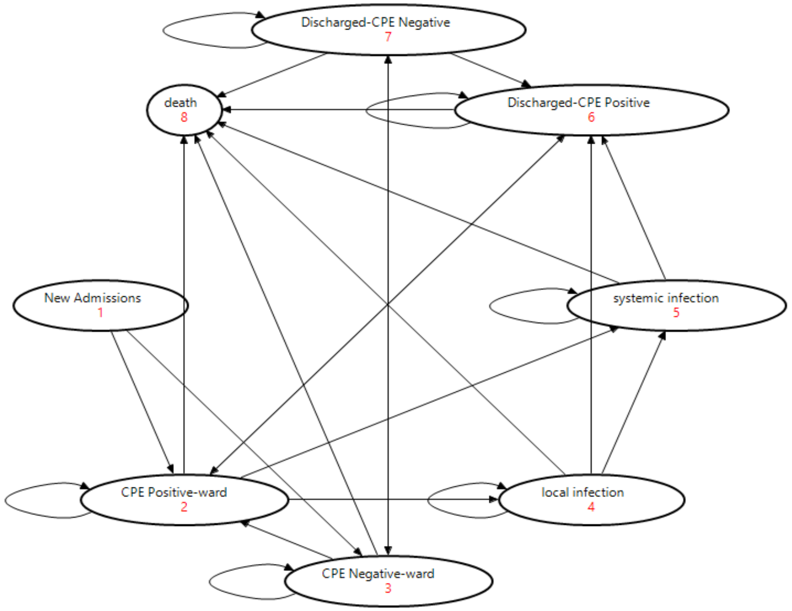

Figure 1 No screen strategy state diagram

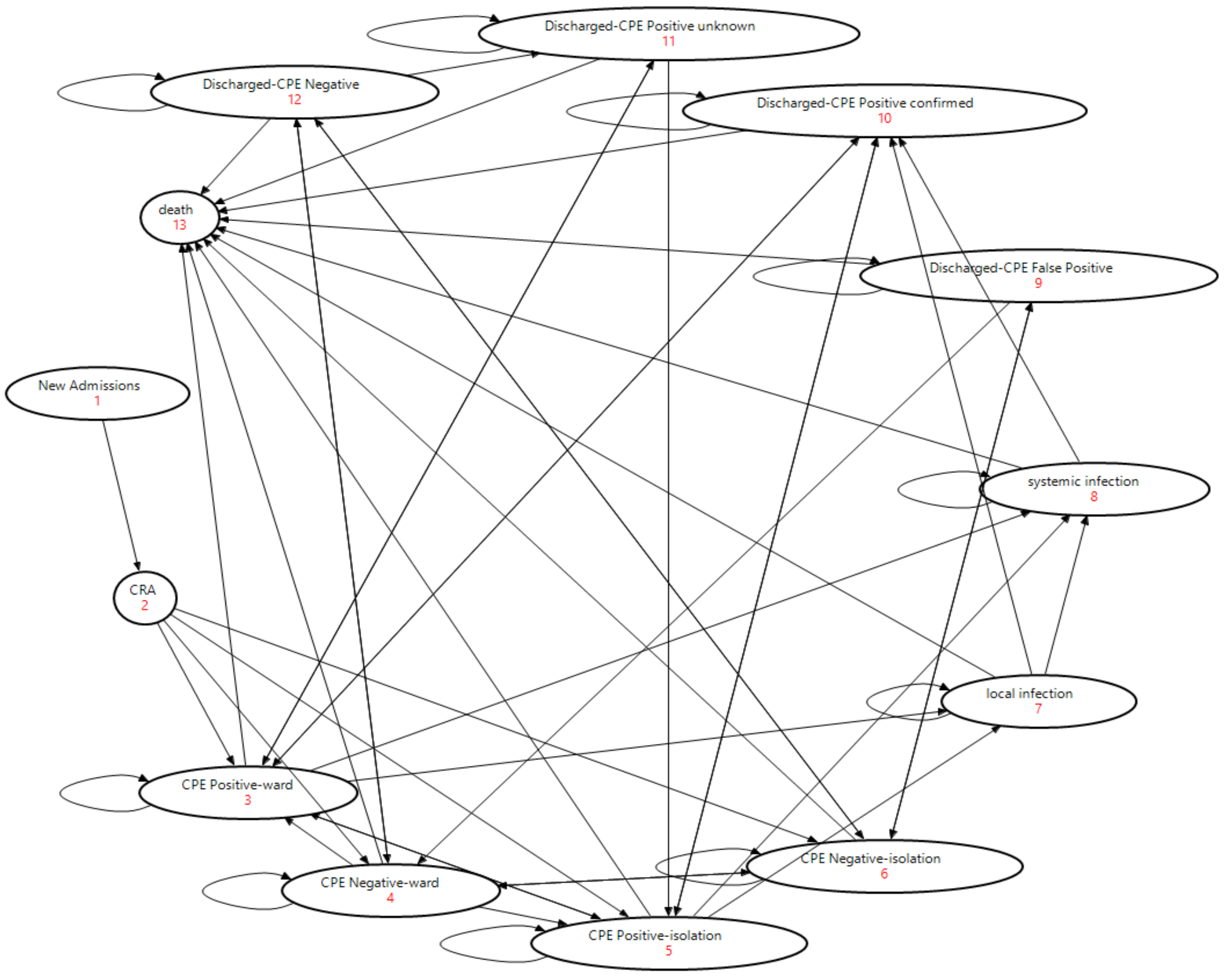

Figure 2 CRA Screening strategy state diagram

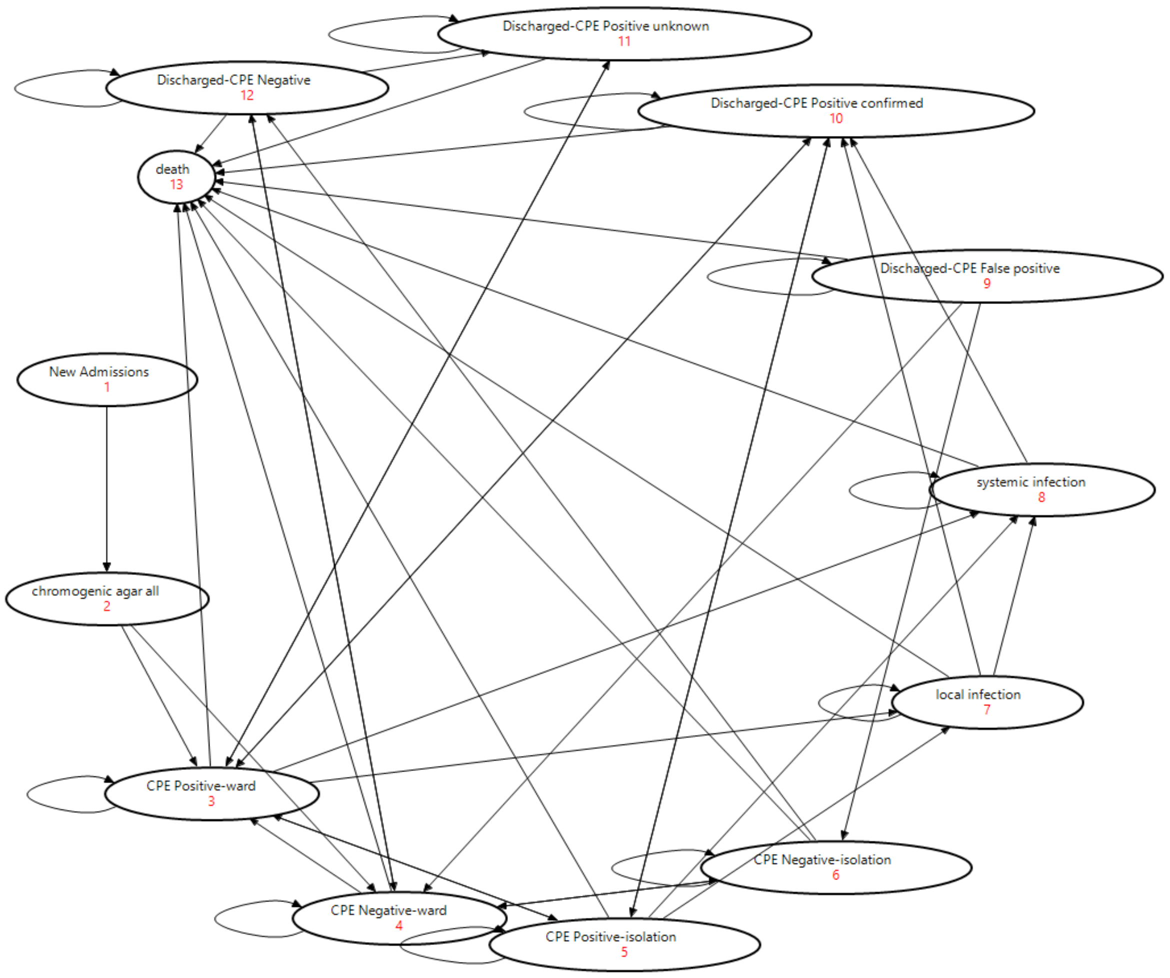

Figure 3 Screen all strategy state transition diagram

2. Model Parameters

Table 1 Model Parameters

Parameter type	Parameter	Description	Value	Plausible Range	Distribution	Source	Notes
Hospital parameters	Age	Age of patient when created in the model	N/A	20-99	N/A	ISD	Model produces a similar age distribution as patients in NHSScotland. Only impact of this parameter is on risk of death from any cause and re-admission risk.
	Bed number	Number of beds in simulated hospital. Based on average beds of teaching and large general hospitals in NHS Scotland	600	N/A	N/A	ISD	The model assumes that patients mix homogeneously in a single hospital ward. This simulates spread of organisms across a hospital through patient and staff movement.
	Daily new admissions	Number of new daily admissions	80	N/A	N/A	ISD	New patients are admitted every day.
CPE Parameters	Basic reproduction number	Average number of secondary cases caused by one typical colonised individual in a population consisting of susceptibles only.	1.1	0.5-3.0	N/A	[1], [2]	"Although the uncertainty surrounding this estimate is important, the steadily increasing number of reports of CPE in hospitals around the world strongly suggests an average basic reproductive rate above 1."<br><br>Values in plausible range are randomly selected in each model iteration.

Parameter type	Parameter	Description	Value	Plausible Range	Distribution	Source	Notes
	pColonised	Probability CPE positive on admission	0.0001	0.0001-0.1	N/A	[3], [4], [5]	Model is run separately for 4 levels of this parameter.
**Daily probabilities**	pLocal	Daily Probability of progression from CPE positive to local infection	0.008078732	0.001919127-0.058868086	Beta	[6]	Beta distribution that approximately results in values shown in range is used.<br><br>Calculation based on 30-days odds 10.8 95%CI(2.8, 41.9) which translates to daily probs value and 95%CI to plausible range.
	pSystemic	Daily probability of progression from CPE positive to systemic infection	0.000413128	0.000198804-0.00149606	Beta	[7]	Beta distribution that approximately results in values shown in range is used.<br><br>Calculation based on 14% (7-28) of CPE colonised developing CPE BSI.
	plocalsystemic	Daily probability of progression from local infection to systemic infection	0.001487112	0.001238918-0.004426329	Beta	[8]	Progression to bacteraemia or sepsis.<br><br>Range based on between 20% and 55% developing systemic infection from CRE local UTI or pneumonia in a period of 6 months.
	pDeath	Daily probability of Death from any cause	Table 2			ISD	Daily probability of mortality from any cause.
	pDischargeColonisedtoReadm	Daily probability to be readmitted if discharged CPE positive	0.011818768			[9]	Base daily probability that was adjusted for age. Adjustment is a ±10%

Parameter type	Parameter	Description	Value	Plausible Range	Distribution	Source	Notes
							change for a every year change from median age. This mean older patients are more likely to be re-admitted than younger patients.
	pDischargetoReadm	Daily probability to be readmitted if discharged CPE negative	0.002287619			[10]	Base daily probability that was adjusted for age. Adjustment is a ±10% change for a every year change from median age. This mean older patients are more likely to be re-admitted than younger patients.
	pLocalInfDeath	Daily probability of death from CPE local	0.003138753	0.002775528-0.003876926		[11]	
	pSystemicInfDeath	Daily probability of death from CPE systemic	0.020089007	0.017993717 - 0.036649362		[12]	
**LOS Parameters**	LOS general patient	LOS when CPE negative	Table 3			ISD	LOS of a general patient in NHS Scotland.
	LOS CPE colonised	LOS when CPE positive	13	10-16	Gamma	Surveillance data held at Health Protection Scotland (HPS)	Gamma distribution around median value for CPE colonised patients. Patients will stay for LOS determined by this distribution days minus anytime already in hospital at time of colonisation. PBPP application, very low numbers.
	LOS CPE Local infection	LOS when CPE Local	19	16-22	Gamma	Surveillance data held at	Gamma distribution around median value for

Parameter type	Parameter	Description	Value	Plausible Range	Distribution	Source	Notes
						Health Protection Scotland (HPS)	CPE Local infection patients. Patients will stay for LOS determined by this distribution days minus anytime already in hospital at time of colonisation. PBPP application, very low numbers.
	LOS CPE Systemic infection	LOS when CPE Systemic	25	22-28	Gamma	Surveillance data held at Health Protection Scotland (HPS)	Gamma distribution around median value for CPE Systemic infection patients. Patients will stay for LOS determined by this distribution days minus anytime already in hospital at time of colonisation. PBPP application, very low numbers.
**Screening parameters**	pCRA	CRA programme uptake	0.80	0.5-0.90		[13]	Values in plausible range are randomly selected in each model iteration.
	pScreen_CRA	Screening uptake if CRA positive	0.90	0.5-0.95		[13]	Values in plausible range are randomly selected in each model iteration.
	pScreen	Screening programme uptake when universal screening	0.75	0.5-0.90		[13, 14]	Values in plausible range are randomly selected in each model iteration.
	chromogenic_sens	Culture test sensitivity	0.90	0.75-0.99	Beta	[15]	Values in plausible range are randomly selected in each model iteration.
	chromogenic_spec	Culture test specificity	0.90	0.76-0.97	Beta	[15]	Values in plausible range are randomly selected in each model iteration.

Parameter type	Parameter	Description	Value	Plausible Range	Distribution	Source	Notes
	chromogenic_time	Time to get results in days	2	1-3 Days	Normal	[16]	Results back in 2 days (50%) or with 25% probability either next day or in three days
	PCR_sens	PCR test sensitivity	0.95	0.80-100	Beta	[17]	Values in plausible range are randomly selected in each model iteration.
	PCR_spec	PCR test specificity	0.99	0.98-100	Beta	[17]	Values in plausible range are randomly selected in each model iteration.
	PCR_time	Time to get results in days	0 (same day)	0-1 Days	Normal	[16]	Results back on the same day or next day. It is more likely results are back on the same day (75%) than next day (25%).
	CRA_sens	CRA questions sensitivity	0.50	0.3-0.9		[3]	Values in plausible range are randomly selected in each model iteration. Determines how many CPE positive patients are missed by CRA.
	CRA_spec	CRA questions specificity	0.90	0.5-0.99		[3]	Values in plausible range are randomly selected in each model iteration. Determines how many CPE positive patients are falsely picked up by CRA (false positives).
	pIsolationFull_confirmed	Risk that no isolation beds are available for microbiologically confirmed CPE positive patient	0.05			Expert opinion	The risk of CPE confirmed patients not finding an isolation bed is very low.
	pIsolationFull_suspected	Risk that no isolation beds are available for suspected CPE positive patient	0.90			Expert opinion	CRA positive awaiting for screening test

Parameter type	Parameter	Description	Value	Plausible Range	Distribution	Source	Notes
**Cost parameters**	c_ward	Daily cost of ward stay	£486.40	SD: £53.80	Gamma	ISD[18]	Daily cost. Weighted average of teaching and general hospitals in NHS Scotland.
	c_ICU	Daily cost of ICU stay	£1800.41	SD: £354.05	Gamma	ISD[18]	Daily cost. Weighted average of teaching and general hospitals in NHS Scotland.
	c_screen_CRA	Total cost of CRA-one off	£6.13	SD: £0.50	Gamma	[19], [20]	
	c_screen_agar	Total cost of chromogenic agar screening-one off	£11.96	SD: £0.50	Gamma	[19], [20]	
	c_screen_PCR	Total cost of PCR screening-one off	£46.09	SD: £1.01	Gamma	[19], [20]	
	c_room_cleaning	Total daily cost of room cleaning	£21.13	SD: £4.96	Gamma	[21]	
	c_IPC	Total daily cost of IPC measures	£42.66	SD: £1.01	Gamma	[21]	Sum of staff and consumables in supporting parameters below
	c_toxic_test_daily	Colistin toxicity daily testing-Daily	£17.29	N/A	N/A	[22]	Average daily cost for ongoing therapy with colistin.
	c_toxic_test_initial	Colistin toxicity test applied first day of treatment-One off	£121	N/A	N/A	[22]	Initial toxicity test cost applied to therapy on the first day of treatment with colistin.
	c_treat_Local	Treatment cost of CPE local infection	£225.15	SD: £30.50	Gamma	[23, 24] Expert opinion	Daily-Includes triple antibiotic therapy
	c_treat_systemic	Treatment cost of CPE systemic infection	£225.15	SD: £30.50	Gamma	[23, 24] Expert opinion	Daily-Includes triple antibiotic therapy
Supporting and derived cost parameters to determine total daily and one off costs		*c_isolation*	*c_ward+c_room_clean+c_IPC (£550.19)*		*Gamma*		*Daily*

Parameter type	Parameter	Description	Value	Plausible Range	Distribution	Source	Notes
		c_local_inf	*c_ward+c_room_clean+c_IPC+c_treat_Local (£775.34)*		*Gamma*		*Daily- Patients who have CPE Local infection are in isolation.*
		c_systemic_inf	*c_ICU+c_room_clean+c_IPC+c_toxic_test_daily+c_treat_systemic (£2089.35)*		*Gamma*		*Daily- Patients who have CPE Systemic infection are in ICU.*
		IPC costs contact precautions	*£0.50*				*Daily*
		IPC staff cost per day	*£42.16*				*Daily*
**Utilities**	util_inpatient	Utility inpatient	0.65	0.60–0.75	Beta	[25]	This utility applies to inpatients irrespective of colonisation status. Does not depend on age.
	util_disch	Utility in discharged	0.85	0.8–0.95	Beta	[26]	Utility of patients who have been discharged irrespective of colonisation status. Does not depend on age.
	util_Local	Utility in local CPE	0.6	0.54–0.73	Beta	[27]	Utility when in CPE local infection. Does not depend on age. Based on MRSA patient utilities, range based on combining values in source.
	util_Systemic	Utility in systemic CPE	0.5	0.25–0.6	Beta	[27]	Utility when in CPE systemic infection. Does not depend on age. Based on MRSA patient utilities but slightly lower. Assumption that CPE systemic would be worse than MRSA for patient.
Modelling variables	disc_rate	Discount rate	0.035			NICE	
	CyclesPerYear	Number of cycles per year	365.25				Used to calculate daily values for utilities or

Parameter type	Parameter	Description	Value	Plausible Range	Distribution	Source	Notes
							anything that is expressed in yearly terms
	startAge	Age at start	Table 4			ISD	Patient Level simulation. Each patient created is allocated an age from a distribution described in Table 4.
	Time_Horizon	Cycles of simulation	1,096				The simulation runs for 1,096 days.

Table 2 All-cause mortality

Age	Daily Probability
20-39	0.000092310
40-59	0.000394951
60-79	0.001228996
80-99	0.003170969

Table 3 Inpatient patient LOS

Age	Expected LOS
20-29	3.0
30-39	3.0
40-49	4.0
50-59	5.0
60-69	6.0
70-79	8.0
80-89	11.0
90-99	15.0
LOS of patients who become CPE positive or develop infection is extended by LOS parameters in Table 1.	

Table 4 Age allocation probability when patient created in simulation

Age	Probability
20-29	0.0067649892
30-39	0.0077894037
40-49	0.0094934396
50-59	0.0154559043
60-69	0.0184102308
70-79	0.0213791728
80-89	0.0165992360
90-99	0.0041076233

Infectious disease modelling

The model allows modelling of spread of CPE in simulated hospital. The probability of becoming CPE colonised depends on the number of other colonised patients in the wards. Isolated patients are not included in this calculation and these patients have a stable colonisation status but can develop infection if CPE true positive. Discharged true colonised patients cannot transmit CPE or develop infection in the community but have a higher risk of re-admission.

In hospital transmission occurs at rate β which is the basic reproductive rate of CPE but transmission depends on the number of colonised patients in the hospital in the following manner: $\beta I/N$, with I representing the number of colonised patients in the wards, and N the total number of patients in the wards.

Therefore, the probability for a susceptible patient to not acquire CPE at a given day is $e^{-\beta I/N}$ and the probability to acquire colonisation is 1- $e^{-\beta I/N}$. The model assumes a fixed basic reproductive rate for all individuals and that patients mix homogeneously in the hospital [5, 16]. It is assumed that CPE colonisation is permanent and a colonised patient remains colonised until death.

Patients who develop CPE local infection remain in isolation but patients with CPE Systemic infection are moved to intensive care unit (ICU). Patients who survive infection are discharged directly into the community and they do not return back to the wards which may not be what happens in reality but since it was applied similarly across all strategies had no impact on model results. Daily probability of death due to CPE infection was calculated from weekly, monthly or yearly mortality rates reported in the literature

3. Additional Results

Table 1 Total costs, total effectiveness and incremental results of models by prevalence of CPE colonised on admission

Prevalence of CPE positive on admission	Strategies	Total cost	Total effectiveness	Incremental Cost	Incremental Effectiveness
1 in 10,000	No screening (Strategy 1)	£493,794,245	77,232	N/A	N/A
1 in 10,000	CRA Culture (Strategy 2)	£494,653,316	77,239	£859,071	7
1 in 10,000	CRA PCR (Strategy 3)	£495,047,155	77,211	£1,252,909	-22
1 in 10,000	Universal culture (Strategy 4)	£498,805,536	77,244	£5,011,291	11
1 in 10,000	Universal PCR (Strategy 5)	£501,870,894	77,237	£8,076,649	4
1 in 1,000	No screening (Strategy 1)	£497,024,269	77,208	N/A	N/A
1 in 1,000	CRA Culture (Strategy 2)	£497,820,186	77,212	£795,917	4
1 in 1,000	CRA PCR (Strategy 3)	£497,972,999	77,219	£948,729	11
1 in 1,000	Universal culture (Strategy 4)	£501,893,597	77,199	£4,869,327	-9
1 in 1,000	Universal PCR (Strategy 5)	£504,897,808	77,220	£7,873,538	12
1 in 500	CRA Culture (Strategy 2)	£501,056,476	77,212	N/A	N/A
1 in 500	No screening (Strategy 1)	£501,271,612	77,196	£215,136	-16
1 in 500	CRA PCR (Strategy 3)	£501,399,306	77,213	£342,830	0
1 in 500	Universal culture (Strategy 4)	£504,810,507	77,200	£3,754,032	-12
1 in 500	Universal PCR (Strategy 5)	£507,932,030	77,200	£6,875,555	-12
1 in 100	CRA Culture (Strategy 2)	£526,776,860	77,044	N/A	N/A
1 in 100	CRA PCR (Strategy 3)	£527,294,585	77,053	£517,726	9
1 in 100	Universal culture (Strategy 4)	£530,296,813	77,085	£3,519,953	40
1 in 100	No screening (Strategy 1)	£533,133,738	77,003	£6,356,878	-41

Prevalence of CPE positive on admission	Strategies	Total cost	Total effectiveness	Incremental Cost	Incremental Effectiveness
1 in 100	Universal PCR (Strategy 5)	£533,390,837	77,054	£6,613,977	10
Table orders strategies by total costs.					

Deterministic analyses based on single model runs and point estimates of parameters were used to select parameters for sensitivity analysis (not shown). In sensitivity analysis we investigate the impact of selected parameters on the cost-effectiveness results with tornado diagrams. The deterministic analyses are not presented due to the nature of the modelled probabilistic processes, e.g. death or discharge, and due to the considerable parameter uncertainty. The usefulness of the tornado diagram lies in that it gives an indication of the relative importance of parameters to the cost-effectiveness results.

To investigate the impact of specific parameters on our results we constructed two tornado diagrams of incremental net monetary benefits (INMB) between: strategy 1 (no screening) with strategy 2 (CRA with culture) and strategy 1 with strategy 3 (CRA with PCR). Tornado diagrams can only show a comparison between two strategies and we chose these to show a comparison between the current NHS policy (with culture and PCR) and the do nothing option. Tornado diagrams study the impact of any number of individual parameters on the cost-effectiveness results, then present them together in a single analysis. An INMB tornado diagram reports the range of INMBs generated for each parameter's uncertainty range. The parameters included in the diagram are the following: Prevalence on admission, specificity of CRA, CRA take up rate, screening uptake rate for CRA positive patients, sensitivity of CRA and screening programme uptake rate. On the graph the blue portion of the bar represents the INMB range when the parameter value is lower than its base case value. The red portion of the bar represents the INMB range when the parameter is higher than its base case value. The variable range is given in parentheses next to each bar on the graph. A third tornado diagram is also presented which shows the impact of these variables on effectiveness (QALYs) across all strategies.

The tornado diagrams show the CPE colonised prevalence on admission has a 63% impact on the results followed by CRA specificity and the CRA take up rate, which have an impact of 25% and 8%, respectively. The screening uptake in the CRA strategies and the sensitivity of CRA have 3% and 2% impact on the results, respectively. Given that prevalence on admission was kept constant in the cost-effectiveness scenarios presented in the main text the parameter with the biggest impact on effectiveness and therefore cost-effectiveness was the specificity of CRA as shown in Figure 6.

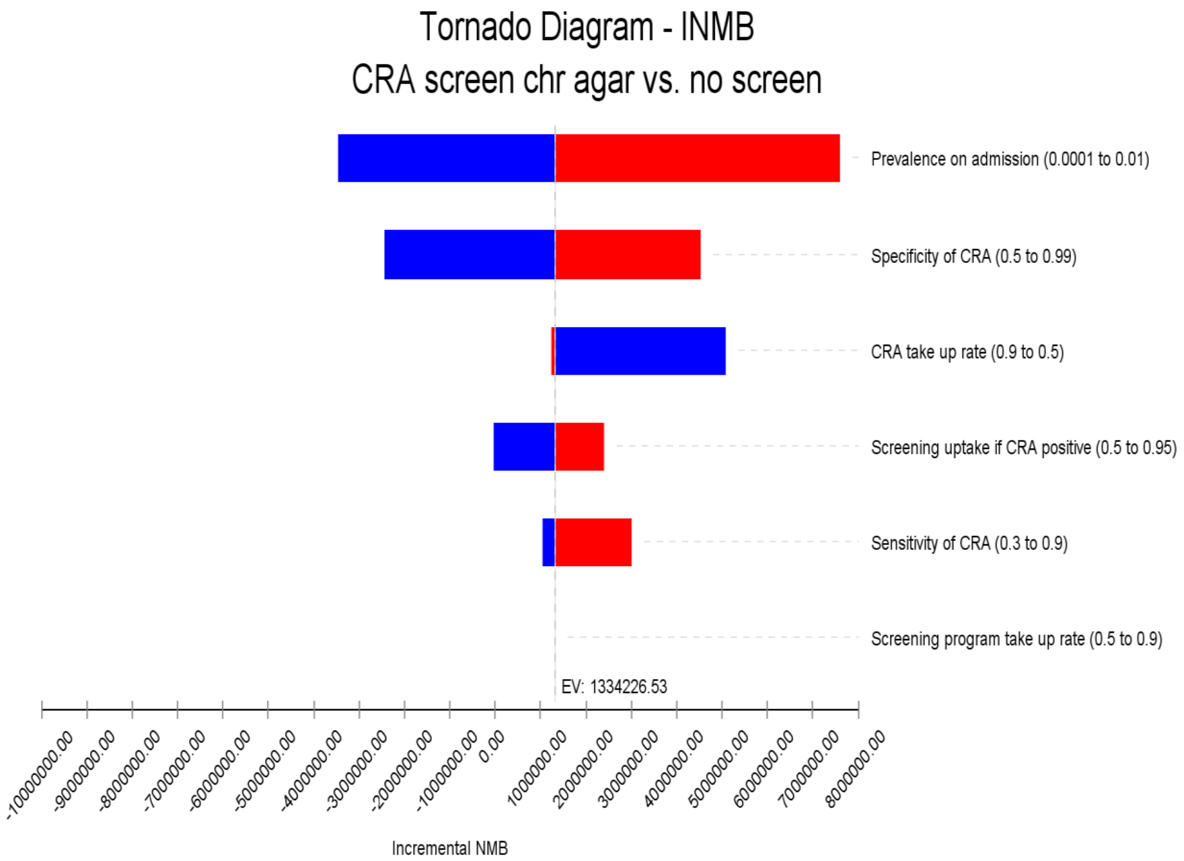

Figure 4 Tornado diagram comparing Strategy 2 (CRA screen chr agar) versus Strategy 1 (no screen)

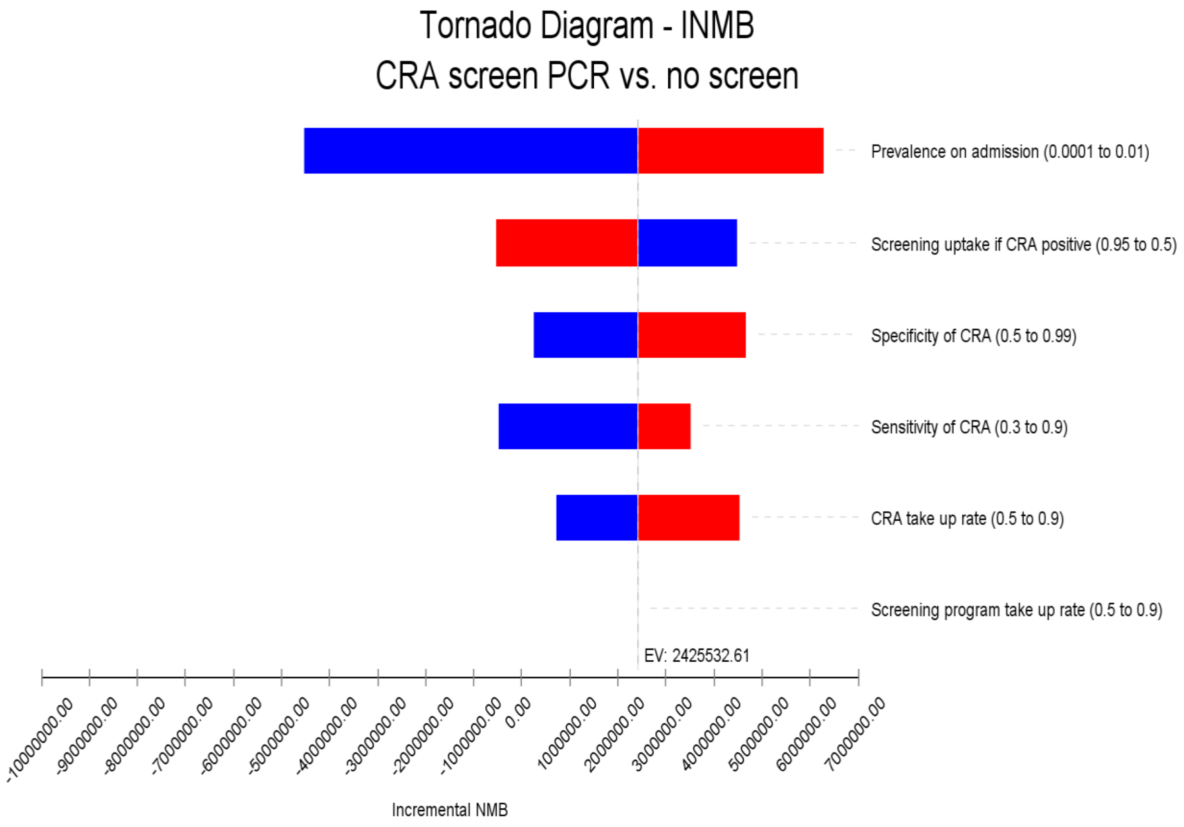

Figure 5 Tornado diagram comparing Strategy 3 (CRA screen PCR) versus Strategy 1 (no screen)

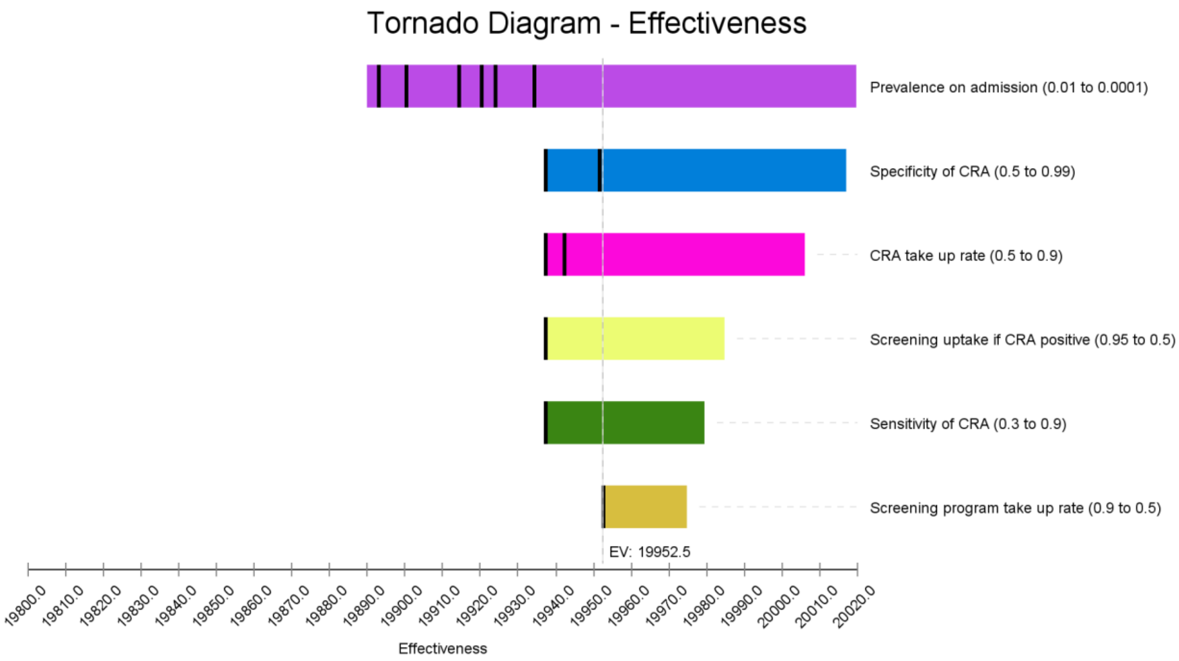

Figure 6 Tornado diagram comparing impact on effectiveness (QALYs) across all strategies

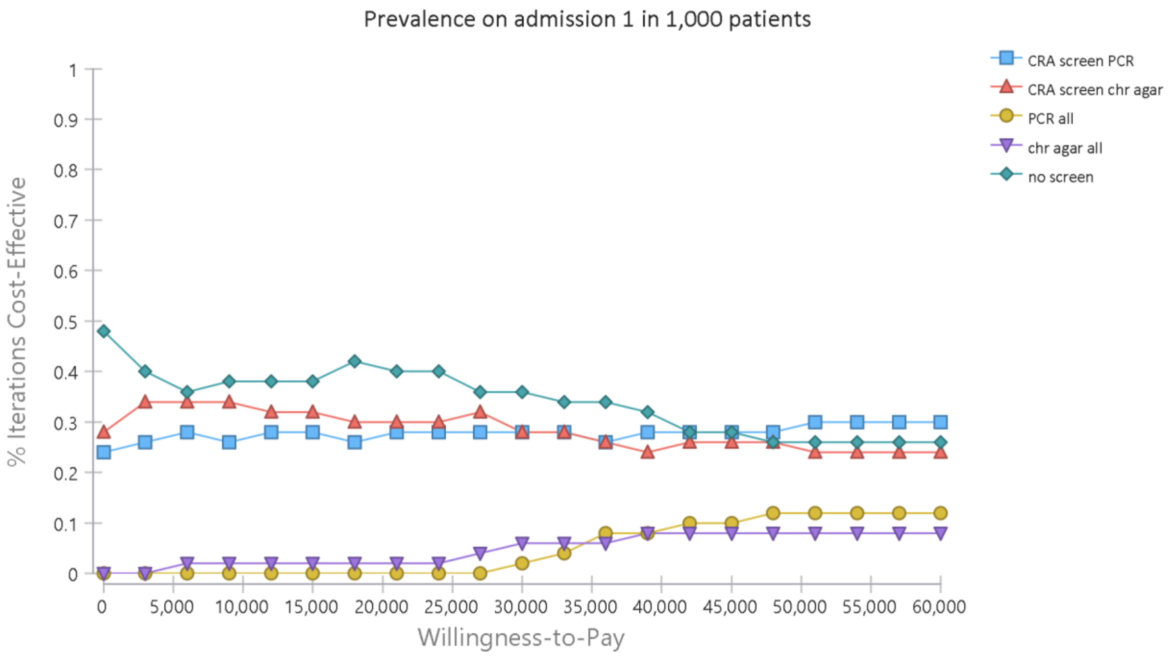

Figure 7 Cost-effectiveness acceptability curve at CPE colonised prevalence on admission 1 in 1,000 patients. "Chr agar" on figure refers to culture microbiological test. "PCR all" and "chr agar all" refers to universal screening strategies.

The results in the scenario of prevalence of 1 in 1,000 patients CPE colonised on admission are shown in Figure 7. In this scenario Strategy 1 "no screening" was the most frequently optimal strategy followed by Strategy 2 (CRA with culture) and Strategy 3 (CRA with PCR). In this scenario these three strategies were very close in terms of the probability of cost-effectiveness. No screening was shown to be the most likely to be cost-effective but the difference with the CRA screening strategies was only a few percentage points. In fact Strategy 3 (CRA screening with PCR) becomes the most likely to be cost-effective when willingness-to pay exceeds £40,000 per QALY gained. The universal screening strategies were not likely to be cost-effective at this level of prevalence exceeding 10% probability over the top of the NHS range of willingness-to-pay per QALY.

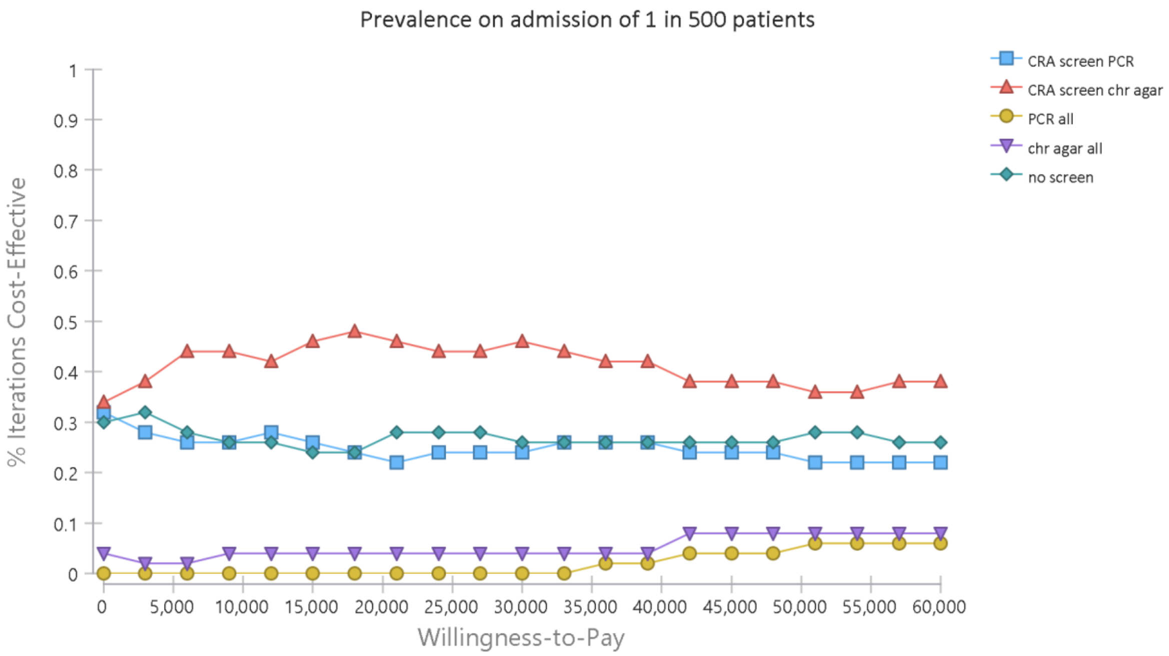

Figure 8 Cost-effectiveness acceptability curve at CPE colonised prevalence on admission 1 in 500 patients. "Chr agar" on figure refers to culture microbiological test. "PCR all" and "chr agar all" refers to universal screening strategies.

The cost-effectiveness acceptability curve in the scenario of 1 in 500 patients being CPE colonised on admission is shown in Figure 8. At this level of prevalence targeted screening becomes cost-saving in relation to no screening. The optimal strategy across the NHS willingness-to-pay range was Strategy 2 (CRA with culture) followed by Strategy 1 (no screening) and Strategy 3 (CRA with PCR). Universal screening was unlikely to be cost-effective in this scenario.

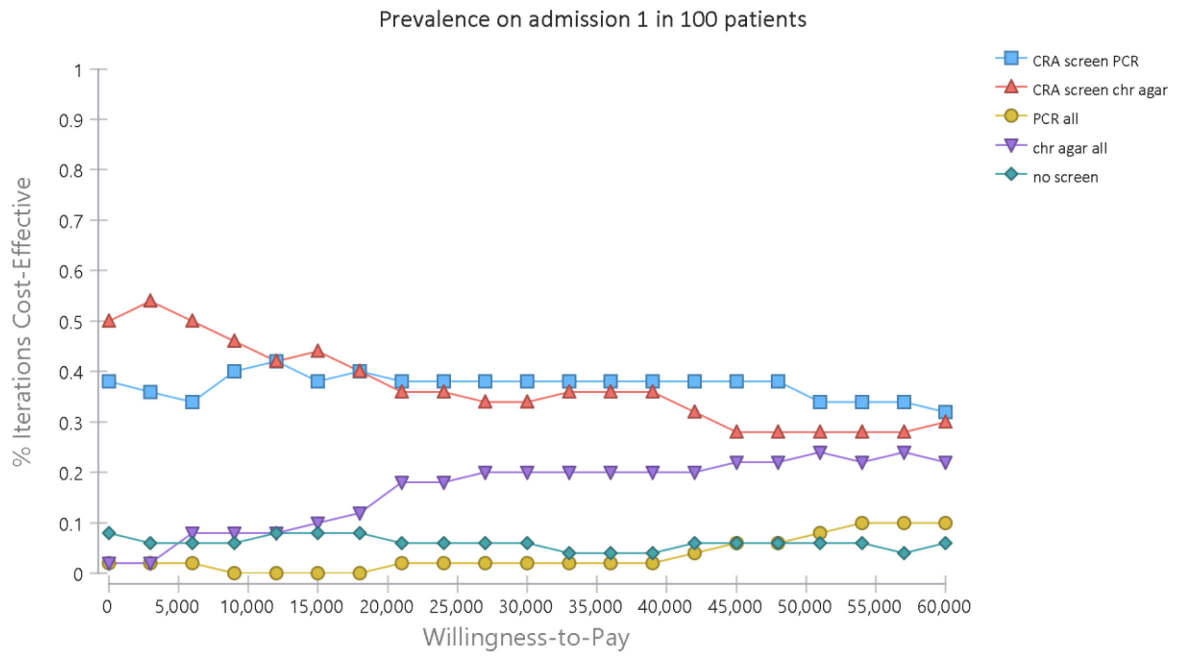

Figure 9 Cost-effectiveness acceptability curve at CPE colonised prevalence on admission 1 in 100 patients. "Chr agar" on figure refers to culture microbiological test. "PCR all" and "chr agar all" refers to universal screening strategies.

The cost-effectiveness acceptability curve in the scenario of 1 in 100 patients being CPE colonised on admission is shown in Figure 9. At this high prevalence CRA screening strategies are by the far the most cost-effective options. Throughout the NHS willingness-to-pay range Strategy 3 (CRA with PCR) was the most cost-effective option very closely followed by Strategy 2 (CRA with culture). This result indicates that the extra speed PCR gives in microbiological test results improves outcomes at this high level of prevalence. It is interesting to note that Strategy 4 (universal screening with culture) had more than 20% probability of cost-effectiveness in the NHS range and approached 30% at higher levels of willingness-to-pay per QALY gained. As CPE positive prevalence on admission increases better coverage given from universal screening became more important to achieve cost-effectiveness. No screening and universal screening with PCR were unlikely to be cost-effective in this scenario.

4. References

1. Haverkate MR, Dautzenberg MJ, Ossewaarde TJ, van der Zee A, den Hollander JG, Troelstra A, et al. Within-Host and Population Transmission of blaOXA-48 in K. pneumoniae and E. coli. PLOS ONE. 2015;10(10):e0140960.
2. Sypsa V, Psichogiou M, Bouzala GA, Hadjihannas L, Hatzakis A, Daikos GL. Transmission dynamics of carbapenemase-producing Klebsiella pneumoniae and anticipated impact of infection control strategies in a surgical unit. PLOS ONE. 2012;7(7):e41068.
3. Otter JA, Dyakova E, Bisnauthsing KN, Querol-Rubiera A, Patel A, Ahanonu C, et al. Universal hospital admission screening for carbapenemase-producing organisms in a low-prevalence setting. JAntimicrobChemother. 2016;71(12):3556-61.
4. Poole K, George R, Decraene V, Shankar K, Cawthorne J, Savage N, et al. Active case finding for carbapenemase-producing Enterobacteriaceae in a teaching hospital: prevalence and risk factors for colonization. JHospInfect. 2016;94(2):125-9.
5. Haverkate MR, Bootsma MC, Weiner S, Blom D, Lin MY, Lolans K, et al. Modeling spread of KPC-producing bacteria in long-term acute care hospitals in the Chicago region, USA. Infect Control Hosp Epidemiol. 2015 Oct;36(10):1148-54.
6. McConville TH, Sullivan SB, Gomez-Simmonds A, Whittier S, Uhlemann AC. Carbapenem-resistant Enterobacteriaceae colonization (CRE) and subsequent risk of infection and 90-day mortality in critically ill patients, an observational study. PLOS ONE. 2017;12(10):e0186195.
7. Tedeschi S, Trapani F, Liverani A, Tumietto F, Cristini F, Pignanelli S, et al. The burden of colonization and infection by carbapenemase-producing Enterobacteriaceae in the neuro-rehabilitation setting: a prospective six-year experience. Infect Control Hosp Epidemiol. 2019 Mar;40(3):368-71.
8. Alexander EL, Loutit J, Tumbarello M, Wunderink R, Felton T, Daikos G, et al. Carbapenem-Resistant Enterobacteriaceae Infections: Results From a Retrospective Series and Implications for the Design of Prospective Clinical Trials. Open Forum Infect Dis. 2017 Spring;4(2):ofx063.
9. Zilberberg MD, Nathanson BH, Sulham K, Fan W, Shorr AF. 30-day readmission, antibiotics costs and costs of delay to adequate treatment of Enterobacteriaceae UTI, pneumonia, and sepsis: a retrospective cohort study. Antimicrob Resist Infect Control. 2017;6:124.
10. Friebel R, Hauck K, Aylin P, Steventon A. National trends in emergency readmission rates: a longitudinal analysis of administrative data for England between 2006 and 2016. BMJ Open. 2018;8:e020325.
11. Zilberberg MD, Nathanson BH, Sulham K, Fan W, Shorr AF. Carbapenem resistance, inappropriate empiric treatment and outcomes among patients hospitalized with Enterobacteriaceae urinary tract infection, pneumonia and sepsis. BMC Infect Dis. 2017 Apr 17;17(1):279.
12. Kohler PP, Volling C, Green K, Uleryk EM, Shah PS, McGeer A. Carbapenem Resistance, Initial Antibiotic Therapy, and Mortality in Klebsiella pneumoniae Bacteremia: A Systematic Review and Meta-Analysis. Infect Control Hosp Epidemiol. 2017 Nov;38(11):1319-28.

13. Reilly JS, Stewart S, Christie P, Allardice G, Smith A, Masterton R, et al. Universal screening for meticillin-resistant Staphylococcus aureus: interim results from the NHS Scotland pathfinder project. JHospInfect. 2010 Jan;74(1):35-41.
14. Cairns S, Packer S, Reilly J, Leanord A. Targeted MRSA screening can be as effective as universal screening. BMJ. 2014 Aug 13;349:g5075.
15. Public Health England. UK Standards for microbiology investigations (UK SMI). London: Standards Unit, Microbiology Services, Public Health England,; 2014 01/08/14.
16. Lapointe-Shaw L, Voruganti T, Kohler P, Thein HH, Sander B, McGeer A. Cost-effectiveness analysis of universal screening for carbapenemase-producing Enterobacteriaceae in hospital inpatients. Eur J Clin Microbiol Infect Dis. 2017 Jan 11;36:1047–55.
17. England PH. Commercial assays for the detection of acquired carbapenemases. London; 2019 30/05/2019.
18. Information Services Division (ISD) Scotland. R04X: Specialty summary - all specialities (excluding long stay), by patient type, by board. 2018 [cited 2019 26 July 2019]; Available from: https://www.isdscotland.org/Health-Topics/Finance/Costs/Detailed-Tables/Speciality-Costs/Acute-Medical.asp
19. Ritchie KB, I.; Craig, J.; Eastget, J.; Foster,L.; Kohli, H.; Iqbal, K.; Macpherson, K.; McCarthy, T.; McIntosh, H.; NicLochlainn, E.; Reid, M.; Taylor, J.; . The clinical and cost effectiveness of screening for meticillin -resistant *Staphylococcus aureus* (MRSA). Health Technology Assessment Report 9. 2007 10/2007.
20. Personal Social Services Research Unit. Unit Costs of Health & Social Care. Canterbury: University of Kent; 2018.
21. Otter JA, Burgess P, Davies F, Mookerjee S, Singleton J, Gilchrist M, et al. Counting the cost of an outbreak of carbapenemase-producing Enterobacteriaceae: an economic evaluation from a hospital perspective. Clin Microbiol Infect. 2017 Mar;23(3):188-96.
22. Antimicrobial Reference Laboratory. Antimicrobial Assay Service - Price list. Bristol: Antimicrobial Reference Laboratory, Department of Medical Microbiology, North Bristol NHS Trust, Southmead Hospital.; 2018 2018.
23. Teare L, Myers J, Kirkham A, Tredoux T, Martin R, Boasman S, et al. Prevention and control of carbapenemase-producing organisms at a regional burns centre. The Journal of hospital infection. 2016 Jun;93(2):141-4.
24. National Institute for Health and Clinical Excellence. British National Formulary. BNF 76. London: BMJ Group; Pharmaceutical Press; 2019.
25. Sackett LD, Torrance WD. The utility of different health states as perceived by the general public. J Chron Dis. 1978;31:697-704.
26. Tengs TO, Wallace A. One Thousand Health-Related Quality-of-Life Estimates. Medical care. 2000;38(6):583-637.
27. Lee BY, Bailey RR, Smith KJ, Muder RR, Strotmeyer ES, Lewis GJ, et al. Universal methicillin-resistant Staphylococcus aureus (MRSA) surveillance for adults at hospital admission: an economic model and analysis. Infect Control Hosp Epidemiol. 2010 Jun;31(6):598-606.
